# Students and Graduates

**Published:** 1850-04

**Authors:** 


### STUDENTS AND GRADUATES.

The University of Pennsylvania numbered four hundred and thirty-eight matriculants.

The Jefferson Medical College, five hundred and sixteen, and two hundred and eleven graduates.

The Pennsylvania Medical College, one hundred and six; thirty-four graduates.

Geneva Medical College, one hundred and six; graduates thirty-two, (1849.)

University of Maryland, one hundred and seventy-one; graduates sixty-eight, (1849.)

Starling Medical College, one hundred and fifty one; graduates fifty, (1849.)

Medical Department of Harvard College, one hundred and twenty-seven.

Medical Department of Yale College, sixteen graduates.

In some instances we have not received the number of graduates for this session; those of 1849 are so designated. At the time of going to press, the session of the University of Pennsylvania is not yet completed, that Institution having extended its lecture term to six months.

